# Supplementary material for: TLR4 Response to LPS Is Reinforced by Urokinase Receptor
Source: Front Immunol. 2020 Dec 9;11:573550. doi: 10.3389/fimmu.2020.573550 (PMC7757075; doi:10.3389/fimmu.2020.573550)
Supplement: Supplementary file 1 [file Table_1.docx]

Supplementary Material

# Supplementary Table 1.

| N | Gene | Producer | Assay ID |
| --- | --- | --- | --- |
| 1 | IL-6 (m) | Applied Biosystems | Mm00446190_m1 |
| 2 | TNFα (m) | Applied Biosystems | Mm00443258_m1 |
| 3 | Cxcl2 (m) | Applied Biosystems | Mm00436450_m1 |
| 4 | MCP-1 (m) | Applied Biosystems | Mm00441242_m1 |
| 5 | GAPDH (m) | Applied Biosystems | Mm99999915_g1 |
| 6 | IL-6 (h) | Applied Biosystems | Hs00174131_m1 |
| 7 | IL-8 (h) | Applied Biosystems | Hs00174103_m1 |
| 8 | GUSB (h) | Applied Biosystems | Hs02786624_g1 |

## Supplementary Figures


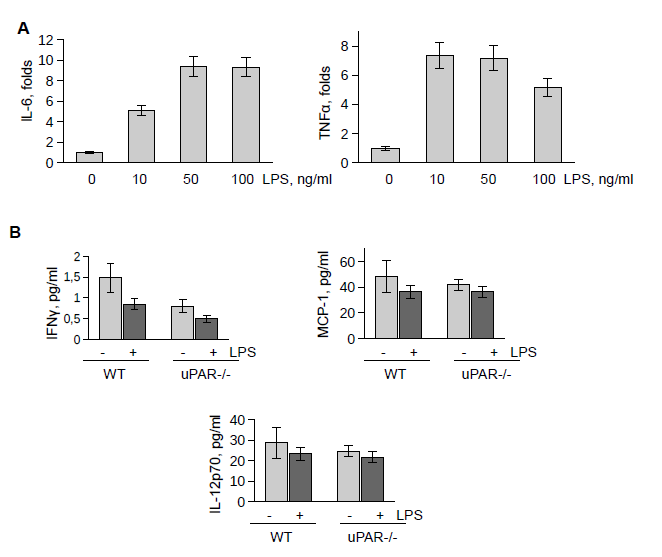


**Supplementary Figure S1.** A. Optimization of the ex vivo mouse blood stimulation with LPS. Mouse blood was stimulated ex vivo with 10, 50, 100 ng/ml LPS for 2 h.IL-6 and TNFα was measured using CBA kit. B. IFNγ, MCP-1, and IL-12p70 measured after ex vivo LPS stimulation of WT and uPAR-/- blood.


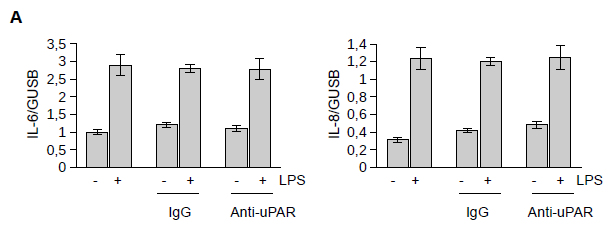


**Supplementary Figure S2.** A. HK-2 were pre-treated with anti-uPAR antibody or IgG before LPS stimulation. Expression of IL-6 and IL-8 was assessed by RT-PCR.


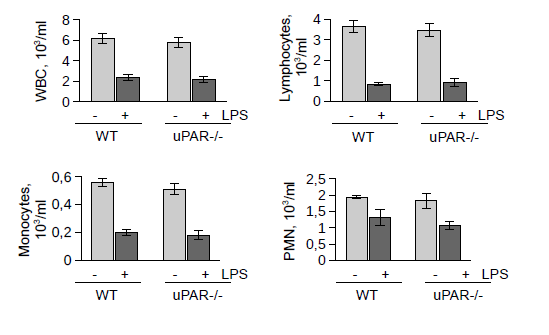


**Supplementary figure S3.** Blood cells count in WT and uPAR-/- 20 hrs after CLP surgery.
